# Exogenously applied spermidine alleviates hypoxia stress in *Phyllostachys praecox* seedlings via changes in endogenous hormones and gene expression

**DOI:** 10.1186/s12870-022-03568-y

**Published:** 2022-04-19

**Authors:** Jianshuang Gao, Shunyao Zhuang, Yuhe Zhang, Zhuangzhuang Qian

**Affiliations:** 1 State Key Lab of Soil and Sustainable Agriculture, Institute of Soil Science, Chinese Academy of Sciences, Nanjing, 210008 China; 2 University of Chinese Academy of Sciences, Beijing, 100049 China

**Keywords:** Flooding, Gene expression, Hormone, Hypoxia, Phyllostachys praecox, Spermidine

## Abstract

**Background:**

Hypoxia stress is thought to be one of the major abiotic stresses that inhibits the growth and development of higher plants. *Phyllostachys pracecox* is sensitive to oxygen and suffers soil hypoxia during cultivation; however, the corresponding solutions to mitigate this stress are still limited in practice. In this study, Spermidine (Spd) was tested for regulating the growth of *P. praecox* seedlings under the hypoxia stress with flooding.

**Results:**

A batch experiment was carried out in seedlings treated with 1 mM and 2 mM Spd under flooding for eight days. Application of 1 mM and 2 mM Spd could alleviate plant growth inhibition and reduce oxidative damage from hypoxia stress. Exogenous Spd significantly (*P* < 0.05) increased proline, soluble protein content, catalase (CAT), superoxide dismutase (SOD), and S-adenosylmethionine decarboxylase (SAMDC) activity, enhanced abscisic acid (ABA) and indole-3-acetic acid (IAA) content, and reduced ethylene emission, hydrogen peroxide (H_2_O_2_), superoxide radical (O_2_^·−^) production rate, ACC oxidase (ACO) and ACC synthase (ACS) to protect membranes from lipid peroxidation under flooding. Moreover, exogenous Spd up-regulated the expression of auxin-related genes *auxin responsive factor1* (*ARF1*), *auxin1 protein (AUX1)*, *auxin2 protein (AUX2)*, *auxin3 protein (AUX3)* and *auxin4 protein (AUX4)*, and down-regulated the expression of ethylene-related *ACO* and *ACS* genes during flooding.

**Conclusion:**

The results indicated that exogenous Spd altered hormone concentrations and the expression of hormone-related genes, thereby protecting the bamboo growth under flooding. Our data suggest that Spd can be used to reduce hypoxia-induced cell damage and improve the adaptability of *P. praecox* to flooding stress.

## Introduction

Hypoxia is a serious impeding factor for plant growth, and results in significant yield losses [1]. Hypoxia mainly includes flooded hypoxia and non-flooded hypoxia, such as soil compaction and mulching [2]. Plants are required oxygen for mitochondrial respiration and energy production. An unanticipated decline in oxygen availability (hypoxia), as caused by roots becoming flooded, or foliage submergence, triggers physiological changes and gene transcription [3]. Flooding stress triggers the production of reactive oxygen species (ROS), which can increase cell membrane permeability, lipid peroxidation and electrolyte leakage [4]. However, the enzymatic defense system composed of different antioxidant enzymes, such as superoxide dismutase (SOD), catalase (CAT), ascorbate peroxidase (APX), and glutathione reductase (GR), actively functions to scavenge ROS and to minimize ROS-caused injuries to biological molecules such as proteins, lipids and nucleic acids [5, 6].

Polyamines (PAs) are a class of low-molecular-weight aliphatic nitrogenous bases with biological activity, that are widely present in bacteria, animals, plants and other living organisms [7]. PAs mainly include diamine putrescine (Put), triamine spermidine (Spd) and tetraamine spermine (Spm), among which Put and Spd exist in all organisms, while Spm only exists in prokaryotic bacteria and eukaryotes [8, 9]. Studies have shown that polyamines can participate in a variety of metabolic processes related to plant growth and development, such as cell division and differentiation, root elongation, leaf senescence, programmed cell death, DNA synthesis, and transcription of genes [10, 11]. The mechanism of the involvement of PAs in plant stress resistance and senescence prevention is that polyamines are closely related to hormones, such as auxin, abscisic acid (ABA) and ethylene which could adjust cell senescence [12–14]. Spd is closely associated with stress tolerance. Previous studies have shown that the application of Spd can enhance plant tolerance to abiotic stresses, such as heavy metals, drought, waterlogging and salt stress [11, 13, 15, 16]. The exogenous application of Spm upregulated the antioxidant systems involving SOD, CAT, APX, GR, glutathione S-transferase (GST), and glutathione peroxidase (GPX) [17]. Yiu et al. [18] reported that Spd and Spm can maintain the water balance of plant leaves and roots under flooding stress. It also significantly delayed the loss of chlorophyll, enhanced photosynthesis, reduced ROS content, and prevented lipid peroxidation caused by flooding. Spd and Spm help maintain the activities of antioxidant enzymes under flooding. The protective effect of Spd was found to be greater than that of Spm [18]. However, the mechanism by which Spd regulates antioxidants under flooding conditions is not yet clear, and it is worthy of further study.

PA is related to many growth and development processes regulated by hormones. Applied with endogenous indole-3-acetic acid (IAA), the content of PAs and key enzymes changed significantly, indicating that there is a synergistic effect of PAs and auxin on plant growth [19, 20]. On the contrary, PAs and ethylene have antagonistic effects, in that PAs inhibit cell senescence and ethylene promotes senescence [21], because PAs and ethylene compete for the common substrate S-adenosyl-L-methionine (SAM). SAM is converted to ethylene by ACC synthase (ACS) and ACC oxidase (ACO) [22]. PAs regulate ethylene biosynthesis by inhibiting the accumulation of ACS transcription, and ethylene is an effective inhibitor of arginine decarboxylase (ADC) and S-adenosylmethionine decarboxylase (SAMDC) [23]. SAMDC is a key enzyme in the synthesis of Spd and Spm [24].

There is a complex network of cross-talk between PAs, ABA and nitric oxide (NO) [11]. For instance, large amounts of ABA are induced to activate downstream gene expression and other physiological responses under flooding [25]. A previous study showed that ABA increased the content of PAs (Put, Spd, and Spm) in grapes and activated the polyamine oxidation pathway, leading to stomatal closure [26]. On the other hand, some studies have shown that ABA has a certain inhibitory effect on the amount of PAs in plant tissues [27]. Alcázar et al. found that PAs regulate stomatal responses by inducing closure and reducing aperture, partly via interaction with ABA and NO [28].

*Phyllostachys praecox* f. is a monoaxial scattered bamboo species of the genus *Phyllostachys* sub-family of *Gramineae*. The shoots of *P. praecox* are delicious in taste, high in nutritional value, early in budding, long in duration, high in yield and low in planting cost. Taking Lin'an City, Zhejiang Province of China as an example, the planting area of *P. praecox* was 3,133 hectares, with an output value of 578 million RMB (almost 1 billion dollar) in 2012. It has been widely promoted and cultivated in most Southern provinces of China [29]. Due to the high level of rainfall in southern China, the roots of the *P. praecox* are susceptible to flooding and thus oxygen deprivation. It is therefore necessary to determine the molecular mechanisms of adaptation to hypoxia and the role of Spd in regulating *P. praecox* hypoxia so that it can be applied in practical production. Therefore, the objectives of this study were to test the following hypotheses: (1) exogenous Spd can alleviate growth inhibition and oxidative damage of *P. praecox* under soil hypoxia stress; and (2) the cross-talk between Spd and hormones triggers the expression of related genes, and initiates downstream protective mechanisms. This study could provide a reference for illustrating the stress resistance mechanism of *P. praecox* under soil hypoxia, and help develop more stress-tolerant varieties to meet a sustainable production.

## Results

### Plant growth effects following flooding

**Fig. 1:**
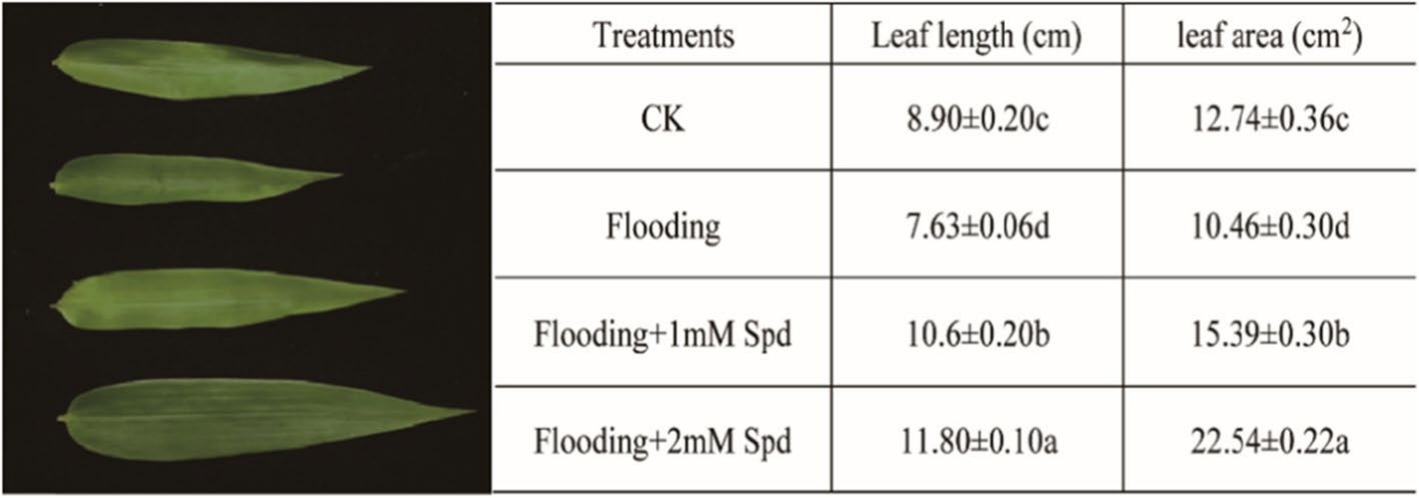
Effects of exogenous Spd application on leaf length and area of *P. praecox* after 8 d of flooding. Vertical bars represent ± the standard error of the mean (*n* = 5, n represents the biological replicates). Values for the same day followed by different letters are significantly different (*P* < 0.05)

The bamboo leaf length (LL) and area (LA) following treatment with Spd are shown in Fig. 1. After 8 d of incubation, the LL and LA were obviously reduced in comparison with the control. Exogenous Spd application alleviated the LL and LA reduction under flooding, and increased the leaf size to some extent (Fig. 1).

### Osmotic adjustment substances

**Fig. 2:**
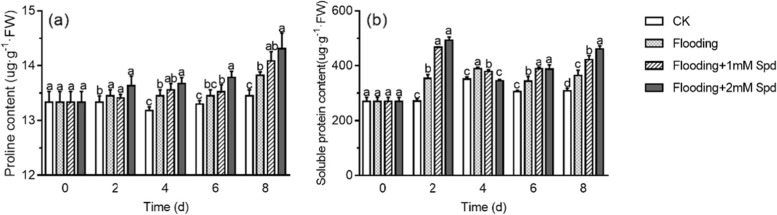
Effects of exogenous Spd application on proline **a** and soluble protein **b** content of leaves in *P. praecox* under flooding for the indicated duration. Vertical bars represent ± the standard error of the mean (*n* = 5, n represents the biological replicates). Values for the same day followed by different letters are significantly different (*P* < 0.05)

After 2 d, the proline and soluble protein content of leaves under flooding condition were significantly increased compared with the control (Fig. 2). And Spd application significantly (*P* < *0.05*) increased soluble protein content compared with that of flooding (Fig. 2b). After 8 days, the proline content and soluble protein content of flooding were significantly improved (*P* < *0.05*) than that of the control. 2 mM Spd significantly increased proline and soluble protein content compared with flooding treatment.

### ROS and membrane damage affected by flooding

**Fig. 3:**
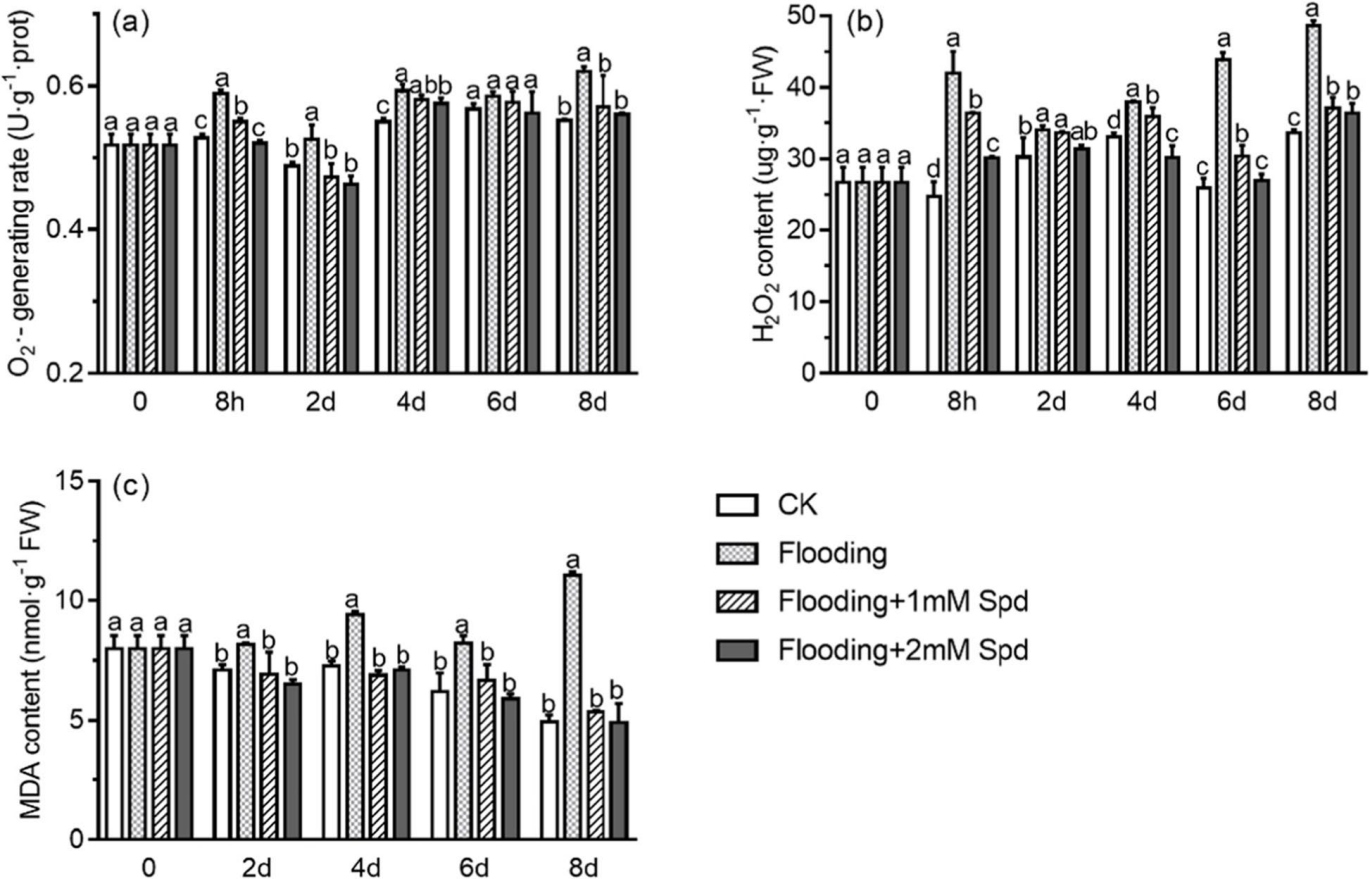
Effects of exogenous Spd application on O_2_^·−^ production rate **a**, H_2_O_2_ **b** and MDA **c** content of leaves in *P. praecox* under flooding for the indicated duration. Vertical bars represent ± the standard error of the mean (*n* = 5, n represents the biological replicates). Values for the same day followed by different letters are significantly different (*P* < 0.05)

After 8 h, flooding significantly (*P* < *0.05*) increased O_2_^·−^ production rate and H_2_O_2_ content by 11.8% and 67.9%, respectively, compared to the control. While exogenous Spd decreased O_2_^·−^ production rate and H_2_O_2_ content under flooded conditions. After 8 d, flooding increased H_2_O_2_ content and O_2_^·−^ production rate compared with the control. However, the addition of Spd significantly (*P* < *0.05*) reduced H_2_O_2_ content and O_2_^·−^ production rate compared with the flooding groups (Fig. 3a, b). The results indicated Spd played a role in controlling ROS homeostasis in flood-stressed plants.

With the extension of flooding time, the concentration of MDA increased and was higher than that of the control. After 8 d of flooding, the MDA concentrations increased by 123.5% (Fig. 3c), compared with control (CK) plants. However, MDA content was also found to be significantly (*P* < 0.05) decreased in flooding + 1 mM Spd (52%) and flooding + 2 mM Spd treatments (18.6%), compared with flooding treatment alone (Fig. 3c). There was no significant difference (*P* < 0.05) between Spd treatment groups and CK. These results indicate that exogenous Spd caused a significant (*P* < 0.05) decrease in the concentration of MDA under flooding treatment.

### Effect of flooding on activity of stress-related enzymes

**Fig. 4:**
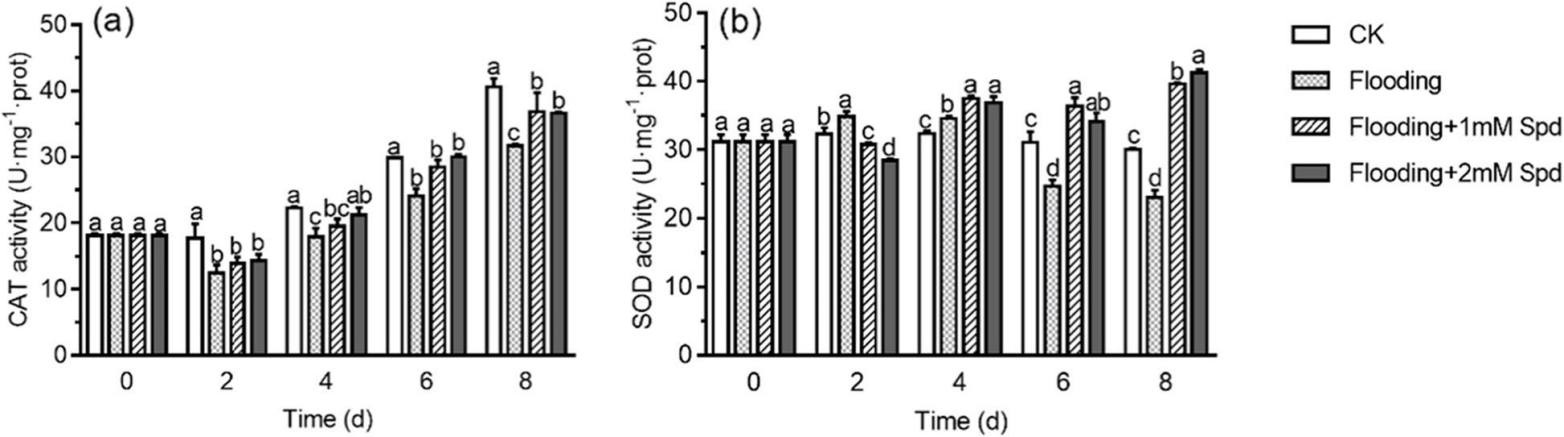
Effects of exogenous Spd application on CAT **a** and SOD **b** activities of leaves in *P. praecox* under flooding for the indicated duration. Vertical bars represent ± the standard error of the mean (*n* = 5, n represents the biological replicates). Values for the same day followed by different letters are significantly different (*P* < 0.05)

After 2 d, flooding significantly (*P* < 0.05) decreased CAT activity but increased SOD activity (Fig. 4). After 8 d, flooding significantly (*P* < 0.05) reduced CAT and SOD activity by 28.2% and 23.3%, respectively. Exogenous Spd significantly (*P* < 0.05) increased the activities of CAT and SOD. Furthermore, with increased Spd concentrations, SOD activity decreased at 8 d (Fig. 4b).

**Fig. 5:**
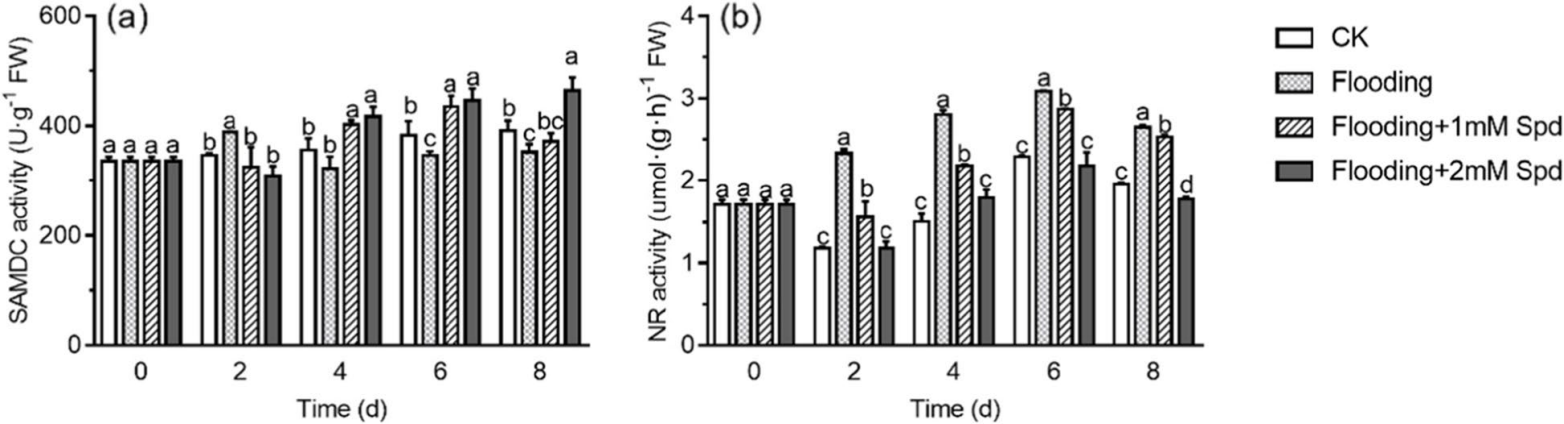
Effects of exogenous Spd application on SAMDC **a** and NR **b** activities of leaves in *P. praecox* under flooding for the indicated duration. Vertical bars represent ± the standard error of the mean (*n* = 5, n represents the biological replicates). Values for the same day followed by different letters are significantly different (*P* < 0.05)

After 2 d, flooding significantly (*P* < 0.05) increased SAMDC activity (Fig. 5a). During the 4–8 days of flooding, SAMDC activity in leaves decreased significantly (*P* < 0.05) under flooding, whereas exogenous Spd application increased the SAMDC activity under flooding (Table 1). There was no significant (*P* < 0.05) difference between flooding + 1 mM Spd and flooding + 2 mM Spd treatments.

**Table 1:** The F value was obtained from the analysis of variance (ANOVA) on the data of the hormone concentration and enzyme activities in the leaves when different concentrations of exogenous Spd were applied under flooding

Source of variation	df	proline	soluble protein	H_2_O_2_	O_2_^·−^ production rate	MDA	CAT	SOD	SAMDC	IAA	ABA	ethylene emission	ACS	ACO
Flooding	1	15.99^**^	449.08 ^***^	251.99 ^***^	67.08 ^***^	388.13 ^***^	162.59 ^***^	25.76 ^***^	2.31^*^	4.46^*^	1.65^*^	314.24 ^***^	289.46 ^***^	49.04 ^***^
time	4	22.69 ^***^	239.09 ^***^	113.67 ^***^	74.62 ^***^	162.02 ^***^	741.41 ^***^	47.94 ^***^	6.89^**^	4.68^**^	41.09 ^***^	27.20 ^***^	58.53 ^***^	12.26 ^***^
1 mM Spd	1	2.81 ns	0 ns	130.87 ^***^	12.29^**^	233.26 ^***^	29.06 ^***^	330.36 ^***^	4.72^*^	75.70 ^***^	51.68 ^***^	76.73 ^***^	177.78 ^***^	8.93^**^
2 mM Spd	1	18.14 ^***^	147.23^***^	278.17^***^	35.79^***^	501.57^***^	110.56^***^	434.11 ^***^	20.34 ^***^	157.95 ^***^	170.23 ^***^	128.54 ^***^	420.54 ^***^	4.82^*^
Flooding × time	4	2.04 ns	90.51 ^***^	43.63 ^***^	8.26^***^	80.12 ^***^	14.23 ^***^	39.46^***^	6.33^***^	19.34^***^	11.50^***^	20.16 ^***^	31.59^***^	6.52^**^
1 mM Spd × time	4	1.12 ns	0 ns	36.60 ^***^	2.40 ns	44.34 ^***^	4.27^*^	164.26 ^***^	8.54^***^	18.26^***^	8.08^**^	5.355 ^**^	16.55^***^	3.28^*^
2 mM Spd × time	4	1.94^*^	54.82 ^***^	41.99 ^***^	4.97^***^	81.56 ^***^	11.89 ^***^	276.55^***^	16.15 ^***^	24.57^***^	16.56^***^	12.02 ^***^	39.63^***^	4.48 ^**^

Nitrate reductase (NR) activity in leaves first increased and then decreased gradually throughout the experimental period following flooding. Compared with the flooded group, NR activity of 1 mM Spd treatment decreased significantly (*P* < 0.05) by 33% on the 2nd, but the decrease rate decreased with the time to 4% on the 8th day. Furthermore, with increased Spd concentrations, NR activity decreased throughout the experimental period (Fig. 5b).

**Fig. 6:**
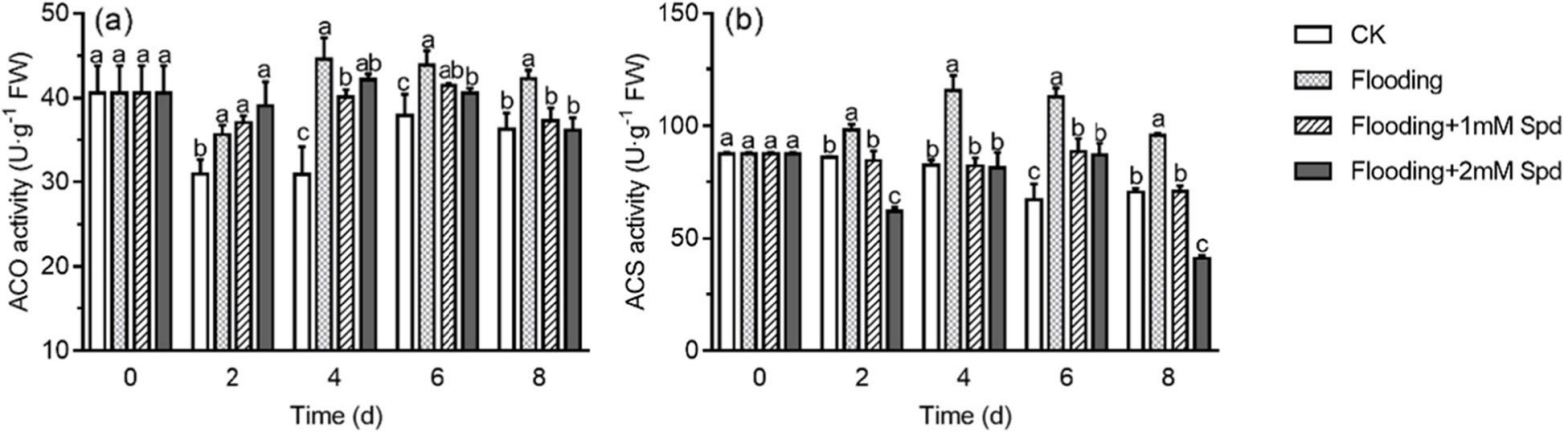
Effects of exogenous Spd application on ACO (a) and ACS (b) activities of leaves in *P. praecox* under flooding for the indicated duration. Vertical bars represent ± the standard error of the mean (*n* = 5, n represents the biological replicates). Values for the same day followed by different letters are significantly different *(P* < 0.05)

ACO and ACS activities of leaves in *P. praecox* under flooding significantly (*P*<0.05) improved (Fig. 6). After 4 d flooding treatment, the ACO and ACS activities were increased by 44.3% and 40.4%, respectively, compared with control plants. Furthermore, as the concentration of Spd increased, the activity of ACS decreased (Fig. 6b). However, no significant difference (*P* < 0.05) in ACO activity was observed in the plants with 1 mM Spd + flooding and 2 mM Spd + flooding treatments (Fig. 6a).

### Effect of flooding on hormone content

**Fig. 7:**
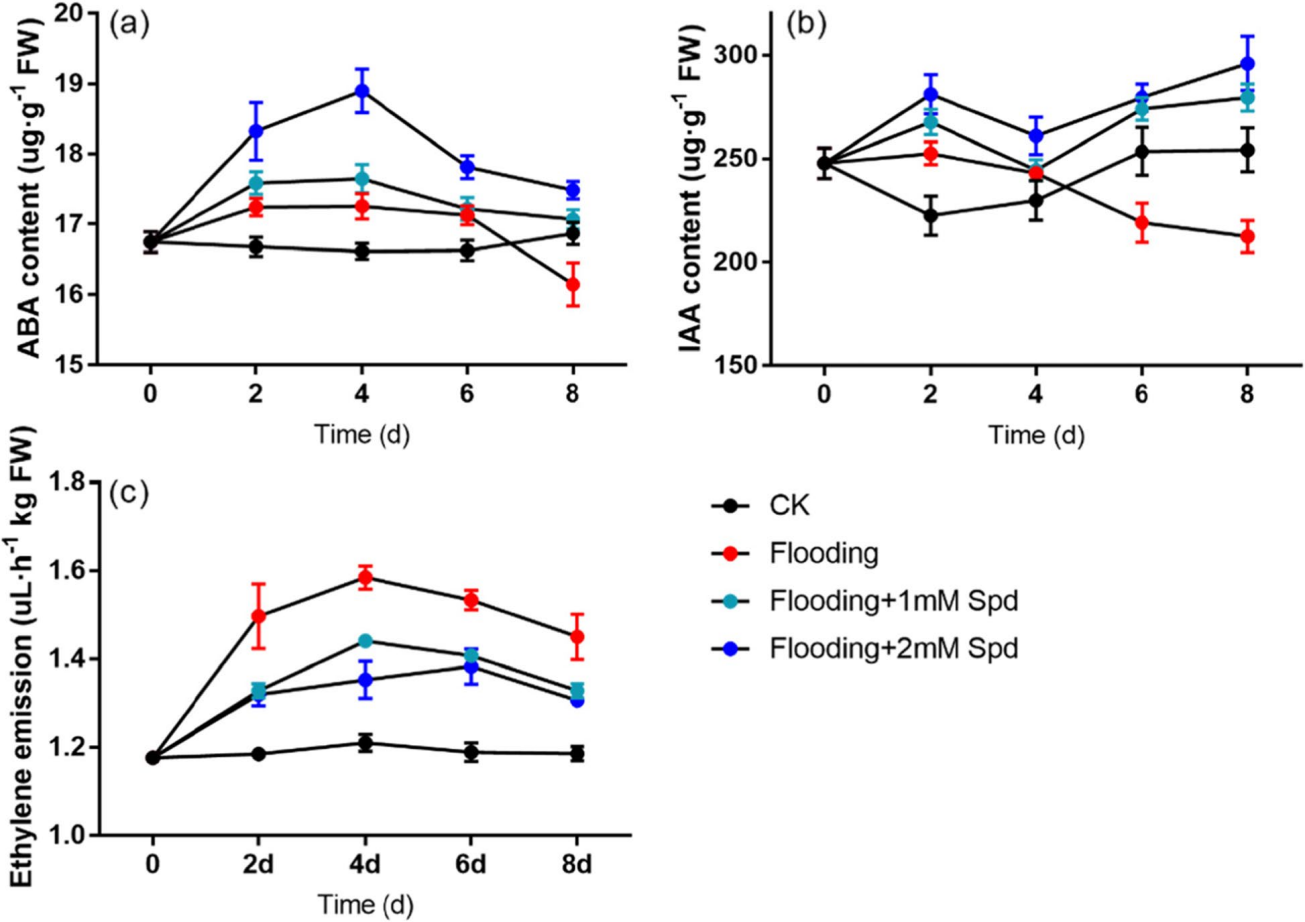
Effects of exogenous Spd application on ABA **a** and IAA **b** content, ethylene emission **c** of leaves in *P. praecox* under flooding for the indicated duration. Vertical bars represent ± the standard error of the mean *(n* = 5, n represents the biological replicates). Values for the same day followed by different letters are significantly different (*P* < 0.05)

Flooding increased ABA content during the 2–6 days of the experiment and exogenous Spd increased ABA content compared to the flooding (Fig. 7a). With the increase in Spd concentration, the ABA content increased as well. After 8 d flooding treatment, the ABA content was significantly (*P* < 0.05) decreased compared with that of control, and Spd improved ABA content to the control level.

After two days of flooding stress, IAA concentrations increased compared with the control (Fig. 7b). Exogenous Spd increased IAA content under flooding. After 8 d flooding treatment, there was an obvious decrease in IAA content compared with the control. However, the IAA concentration was increased by Spd treatment, and it increased with the increase in Spd concentration.

Flooding increased ethylene emission during the experiment, but exogenous Spd decreased ethylene emission (Fig. 7c). The ethylene emission of leaves increased first and then decreased with time. After 8 d of flooding treatment, the ethylene emission was significantly (*P* < 0.05) increased compared with that of the control, and Spd application significantly (*P* < 0.05) reduced it under flooding. There was no significant difference between 1 mM Spd and 2 mM Spd treatment under flooding conditions.

### Effect of flooding on relative gene expression

**Fig. 8:**
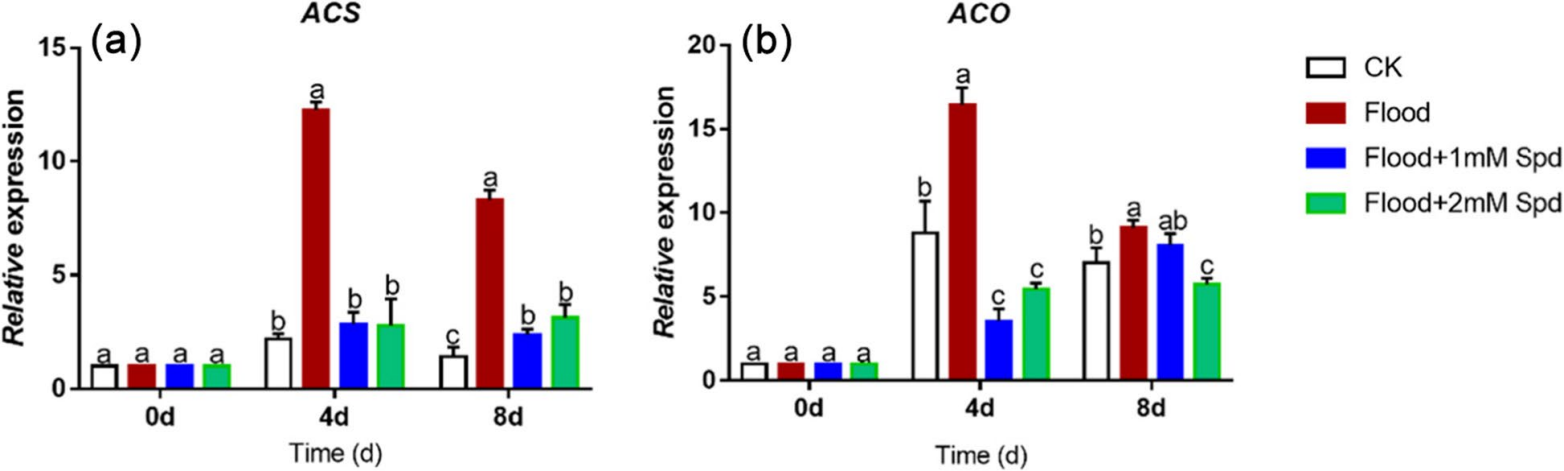
Effects of exogenous Spd application on the relative expression of *ACS* **a** and *ACO* **b** in leaves of *P. praecox* under flooding for 0, 4 d and 8 d. Vertical bars represent ± the standard error of the mean (*n* = 5, n represents the biological replicates). Values for the same day followed by different letters are significantly different (*P* < 0.05)

Expression of *ACS* and *ACO* first increased and then decreased during the course of the experiment (Fig. 8). After 4 d of flooding treatment, expression of *ACS* and *ACO* in leaves under flooding were significantly (*P* < 0.05) up-regulated to 87.3% and 29.6% of the controls, respectively. On the other hand, exogenous Spd treatment down-regulated *ACO* and *ACS* gene expression after 4 d and 8 d flooding treatment.

**Fig. 9:**
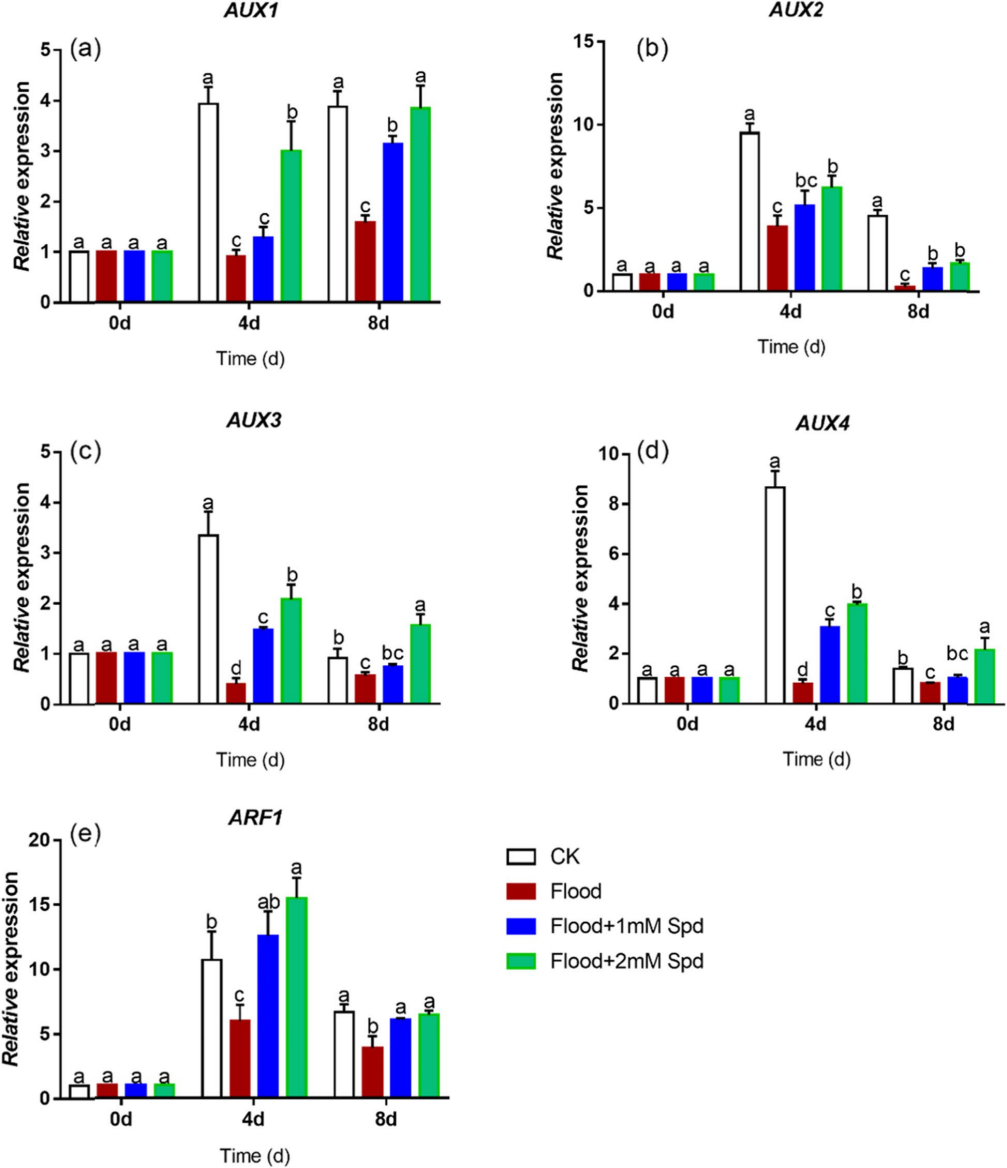
Effects of exogenous Spd application on the relative expression of *AUX1* **a**, *AUX2* **b**, *AUX3* **c**, *AUX4* **d**, and *ARF1* **e** in leaves of *P. praecox* under flooding for 0, 4 d and 8 d. Vertical bars represent ± the standard error of the mean (*n* = 5, n represents the biological replicates). Values for the same day followed by different letters are significantly different (*P* < 0.05)

Expression of *AUX2*, *AUX3* and *AUX4* increased and then decreased during the experiment. After 4 d flooding treatment, expression of *AUX1*, *AUX2*, *AUX3,* and *AUX4* was significantly (*P* < 0.05) reduced to 76.9%, 59.1%, 88.3%, and 90.6% compared with the controls, respectively (Fig. 9a-d). Treatment with flooding + 2 mM Spd significantly (*P* < 0.05) up-regulated expression of *AUX1* and *AUX2,* and 1 mM Spd did not alter the expression under flooding at 4 d (Fig. 9a, b). After 8 d, with the increase in Spd concentration, *AUX1* expression was also up-regulated (Fig. 9a). There was no significant (*P* < 0.05) difference in *AUX2* expression between flooding + 1 mM and flooding + 2 mM Spd plants (Fig. 9b). Exogenous Spd at 2 mM up-regulated the expression of *AUX3* and *AUX4* under flooding. Treatment with 1 mM Spd did not significantly (*P* < *0.05*) alter the expression of *AUX3* and *AUX4* under flooding after 8 d (Fig. 9c, d).

In the control treatments, expression of *ARF1* first increased and then decreased. Flooding caused a sharp down-regulation in *ARF1* expression, when compared with the control. After 4 d flooding treatment, the expression of *ARF1* was down-regulated by 44.8% compared with control (Fig. 9e). After 8 d, *ARF1* expression decreased overall. However, exogenous Spd up-regulated the expression of *ARF1* under flooding and reached the control levels. There was no significant (*P* < 0.05) difference between expression of *ARF1* in flooding + 1 mM Spd and flooding + 2 mM Spd treatments.

## Discussion

As aerobic organisms, plants need oxygen (O_2_) to support respiration, metabolism and growth. Plants frequently suffer from hypoxic stress due to the low O_2_ concentration that is caused by long-term flooding, waterlogging, soil compaction or soil cover management [2, 30]. Hypoxia triggers plant physiological changes and gene expression [3]. In our study, flooding was shown to inhibit the leaf length and leaf area in *Phyllostachys praecox,* as expected. Exogenous Spd alleviated bamboo growth inhibition with flooding (Fig. 1). This was consistent with the result that Spd alleviated the inhibition of soybean seedling growth under excess soil moisture [31]. Zhang et al. also found exogenous Spd attenuated iso-osmotic salt stress-induced reductions in growth parameters [32].At the same time, in order to adapt to flooding stress, plants would synthesize proline, soluble protein and other osmotic adjustment substances in a short period of time [33]. In the present study, proline and soluble proteins content were markedly increased after flooding. 2 mM Spd increased the content of proline and soluble protein furthermore (Fig. 2; Table 1). Because enhanced proline was a scavenger of ·HO and singlet oxygen, it can reduce stress-induced cell acidification to protect cell membrane and metabolic function [34, 35]. Tanou et al. [36] suggested that high proline content achieved by polyamine application results from the changes in its gene expression.

Flooding triggered overproduction of ROS, whose accumulation causes indirect or direct oxidative damage to the plants exposed to stresses, thus causing significant damages to cellular constituents, especially membrane lipids [37, 38]. Here we also found that flooding stimulated plants to produce more ROS, such as H_2_O_2_ content and O_2_^·−^ generation rate (Fig. 3a, 3b; Table 1). Interestingly, exogenous Spd increased H_2_O_2_ content after 4d of flooding. This may be because Spd induced the expression of RBOH (respiratory burst oxidase homolog) and the accumulation of H_2_O_2_ in leaves. The same phenomenon has been found in cucumbers. Zhang [39] found that RBOH-dependent H_2_O_2_ mediated the Spd-induced autophagy and salt tolerance in cucumber. With time under flooding, O_2_^·−^ generation rate, H_2_O_2_ and MDA content increased. The application of Spd reduced the MDA content and ROS lever, indicating that Spd can reduce the oxidative stress caused by flooding (Fig. 3; Table 1). This is in accordance with that reported by Hussain et al. [40], where Spd reduced MDA content and ROS concentrations in *Brassica juncea* leaves. The MDA content is a marker of cell membrane lipid peroxidation. Hence, the increases in MDA content of flooding indicates the presence of oxidative stress derived from the overproduction of ROS [41]; Spd supple-mentation reduced lipid peroxidation [42]. To eliminate excessive H_2_O_2_ and O_2_^·−^, plants regulate SOD and CAT activities to protect against flooding stress [43]. SOD is the only enzyme able to remove O_2_^·−^ [44]. The main role of CAT is to remove H_2_O_2_, which CAT converts H_2_O_2_ to H_2_O [45]. We found that SOD activity increased first and then decreased after flooding, which was due to self-defense mechanism. Flooding significantly decreased CAT activity, but CAT and SOD activities were significantly improved after Spd treatment (Fig. 4; Table 1). It may be that Spd regulated enzymatic activities by scavenging free radicals. The results were consistent with those of alfalfa [43]. Spd application enhanced SOD and CAT activities in alfalfa under salt stress, so as to maintain low MDA and ROS levels, thus alleviating cell damage caused by salt stress.

SAMDC is the rate-limiting enzyme in the synthesis of Spd and Spm in plants [24]. SAMDC alleviates the negative effects of salt stress by inhibiting chlorophyll degradation, maintaining the integrity of chloroplast structural, enhancing the photosynthetic rate and increasing the capacity of the enzymatic and non-enzymatic antioxidant system to mitigate oxidative damage [39, 46, 47]. Studies have reported that after 6 h of drought stress, the PA content in *CaSAMDC*-overexpressing transgenic Arabidopsis increased, and the accumulation of ROS in cells decreased significantly [48]. In the present study, flooding induced an increase in SAMDC activity after 2 d. In the middle and late stages of the experiment, SAMDC activity decreased significantly (*P* < 0.05) under flooding (Fig. 5a; Table 1). The transient increase in SAMDC activity may lead to an increase in PA content to protect bamboo from flooding stress. However, as the degree of stress increases, a large amount of ethylene is synthesized in plants. Therefore, it is possible that the concentrations of Spd and Spm that share a common precursor (SAM) with ethylene decreased, and the activity of SAMDC therefore decreased. We found that after exogenous Spd application, the SAMDC activity increased significantly (*P* < 0.05) and the ACS and ACO activities and gene expression decreased. This may be because exogenous Spd led to an increase in the content of endogenous Spd and Spm in bamboo. The increase in SAMDC activity may accelerate the conversion of free Put to Spd and Spm, and then the conversion of free Spd to Spm, while reducing the ethylene content. This hypothesis is consistent with previous studies, where Hu et al. [49] found that exogenous Spd significantly improved Spd and Spm content, and enhanced SAMDC activity under salt stress.

Nitric oxide plays a key role as an intra- and intercellular messenger, inducing various processes in plants, including the expression of related genes and programmed cell death, stomatal closure, seed germination, cadmium toxicity and root development [50, 51]. The source of NO in plants is very rich, and it is mainly produced through the activities of NO synthase (NOS) and nitrate reductase (NR). NR is a cytosolic enzyme that catalyzes NADH-dependent nitrate reduction into nitrite. Nitrate reduction may contribute to cellular acclimation to low oxygen deprivation by regenerating NAD^+^ from NADH. Accordingly, species tolerant to oxygen deprivation exhibit higher NR activity than sensitive ones [30]. In the present study, we found that flooding significantly (*P* < 0.05) improved the NR activity of leaves (Fig. 5b, Table 1). Both in tobacco and in tomato, it has been shown that the absence or the decrease in NR activity in transgenic plants or the addition of tungstate (a potent inhibitor of NR activity) enhances the symptoms of hypoxia. These symptoms are accompanied by a reduction in plant growth [52, 53]. We also found that exogenous Spd decreased NR activity under flooding conditions. Furthermore, with the increase of Spd concentration, NR activity decreased significantly (*P* < 0.05) (Fig. 5b, Table 1). It might be possible that PAs promote the interaction between NO and 14–3-3 proteins to inhibit NR [54]. Mahadi [55] found that Spm-induced NO is involved in *HSP70* expression, which may function in HSP accumulation, stabilisation of denatured proteins and accurate protein-folding in wheat seedlings under Se-induced oxidative stress.

Polyamines are often regarded as second messengers of plant growth regulators or plant hormones [56]. S-adenosylmethionine (SAM) is a common precursor in the biosynthesis pathway of polyamines and ethylene. Therefore, there may be different interactions between polyamines and ethylene in cells [23]. One possible interaction is the mutually antagonistic relationship, and the other is that there is no antagonistic relationship between the two. ACS and ACO activities are generally the rate-limiting step in the ethylene biosynthetic pathway [57]. In the present study, we found that flooding significantly (*P* < 0.05) increased ethylene emission, enzyme activities and expression of *ACS* and *ACO,* which were significantly (*P* < 0.05) decreased by Spd (Fig. 6, 7c, 8; Table 1, 2). Moreover, ACS is more sensitive to flooding stress than ACO (Table 1, 2). It indicates that Spd regulated ethylene synthesis to mitigate flooding damage. These results are similar to those of previous studies [58, 59]. Polyamines are involved in the regulation of the expression of *ACS* [60]. The production of ethylene can be affected by the regulation of ACC synthase and ACC oxidase; at the same time, ethylene can also affect the amount of polyamines in tissues by inhibiting the activity of polyamine synthases such as ADC [23].

**Table 2:** The F value is obtained from the analysis of variance (ANOVA) on the data of the relative gene expressions in the leaves when different levels of exogenous spd were applied under flooding

Source of variation	df	*ACS*	*ACO*	*ARF1*	*AUX1*	*AUX2*	*AUX3*	*AUX4*
water	1	1583.22^***^	49.40^***^	16.67^**^	344.76^***^	319.37^***^	113.11^***^	352.88^***^
time	2	240.66^***^	343.05 ^***^	245.44^***^	189.58^***^	452.16^***^	81.59^***^	343.17^***^
1 mM Spd	1	1092.56^***^	253.07^***^	37.40^***^	104.64^***^	11.71^**^	206.75^***^	129.63^***^
2 mM Spd	1	320.34^***^	397.77^***^	87.60^***^	97.97^***^	40.37^***^	145.08^***^	229.10^***^
water × time	2	438.82^***^	24.52^***^	5.83^*^	90.95^***^	84.45^***^	81.05^***^	228.03^***^
1 mM Spd × time	2	315.96^***^	199.93^***^	16.50^***^	55.89^***^	2.96^**^	129.04^***^	99.35^***^
2 mM Spd × time	2	101.06^***^	183.31^***^	44.06^***^	24.60^***^	11.98^***^	43.26^***^	84.34^***^

ABA has regarded as one of the main internal plant signals that trigger the various acclimations that plants undergo when exposed to flooding [61, 62]. ABA seems to play a predominant role in the conversion of environmental signals into changes in the gene expression of plants [63, 64]. On the other hand, increasing evidence indicates that ABA interacts with membrane phospholipids to stabilize the membranes under stress conditions [65, 66], and it plays a role in the enhancement of tolerance to oxidative stress by increasing the activity of antioxidant enzymes [67, 68]. In the study, ABA content increased in the early stages of flooding. Exogenous Spd increased ABA content under flooding stress in the initial stages. With the increase of exogenous Spd concentration, the ABA content also increased (Fig. 7a; Table 1). This is consistent with previous studies. Tajti et al. [69] also found that ABA accumulation in Spd-treated plants. In addition, after 8 d flooding, ABA content of flooded plants significantly (*P* < 0.05) decreased compared with controls (Fig. 7a; Table 1). This is probably due to the increase in membrane permeability and cell function damage by the prolonged flooding time. The rate of ABA catabolism was higher than its rate of synthesis, which led to a decrease in the ABA content. It is consistent with previous studies. For example, ABA was also found to decrease greatly in the roots of tomato and Ricinus communis under soil flooding [70, 71].

It is well known that IAA can promote plant growth. At the beginning of flooding, the content of IAA increased in bamboo tissues. The increase of auxin content at the early stage of flooding is beneficial to the initiation of stem elongation, leaf growth and oxygen transport [72, 73]. Similarly, we found that IAA content increased during 2–4 d of flooding. However, we also noted a significant (*P* < 0.05) decrease in IAA content on the sixth and eighth day after flooding (Fig. 7b; Table 1). This is probably because in the late stages of flooding, photosynthesis of *P. praecox* was severely blocked, membrane lipid peroxidation was severe, and cellular structures were destroyed; thus, the bamboo could not provide energy and substances to meet the needs of IAA synthesis. In our study, exogenous Spd significantly (*P* < 0.05) increased IAA content of bamboo under flooding (Fig. 5b; Table 1). This may be a protection mechanism for plants to adapt to the flooded environment. Li et al. [74] suggested that drought stress significantly increased the ABA, methyl jasmonate (MeJA) and salicylic acid (SA) concentrations, and notably decreased the IAA, gibberellins (GA3) and zeatin-riboside (ZR) concentrations in maize seedlings.

Many auxin-related genes participate in plant development by regulating the auxin balance in processes such as cell division and elongation, morphogenesis of roots and stems, apical dominance, and plant leaf bud and fruit development [75, 76]. Some genes, such as auxin/indole-3-Acetic Acid (*Aux/IAA*) are responsive to auxin stimulation in the early stage of auxin signal transduction [77]. Auxin signaling involves the regulation of gene expression by AUXIN RESPONSE FACTORS (ARFs) and their inhibition by Aux/IAA proteins [78]. ARFs can initiate or inhibit the expression of primary early auxin response genes by specifically binding to the auxin response elements (AuxREs) in the promoter part [79], and participate in different growth processes of plants. In Arabidopsis, the ARF protein family is divided into two categories: transcription activator and transcription repressor [80]. So far, only ARF2, ARF3, ARF4 and ARF9 proteins have been proved to have transcriptional inhibition through plant protoplast transformation experiments [81].

There is evidence that the expression of *ARF* is affected by environmental or hormonal signals. For example, as the degree of leaf senescence deepens, the expression of *ARF2* increased, while that of *ARF1* decreased in Arabidopsis leaves [82]. Similarly, we found that flooding significantly (*P* < 0.05) decreased *ARF1* expression of leaves and Spd application up-regulated *ARF1* gene expression under flooding (Fig. 9e; Table 2). It was suggested that *ARF1* was likely to be a transcription activator. It has been reported that *AUX1* is an auxin uptake carrier [83]. *AUX1* could directly be involved in induction of ROS signaling via the H_2_O_2_-mediated pathway, which prevents further increase in oxidative damage. Alternatively, AUX1 could indirectly influence cell elongation and cell division by regulating auxin levels and the auxin signaling network, in turn controlling the root growth under stress [84]. The present study showed that the expression of *AUX1*, *AUX2*, *AUX3* and *AUX4* genes of bamboo under flooding was significantly (*P* < 0.05) reduced, and *AUX1*, *AUX2* and *AUX4* were more sensitive to flooding (Table 2), while exogenous Spd up-regulated the expression of these genes (Fig. 9a-d; Table 2). This may be due to the synergistic effect of spermidine and IAA to alleviate the damage caused by flooding stress.

## Conclusions

Soil hypoxia induced the growth and membrane lipid injury in *P. praecox* leaves. Exogenous application of Spd enhanced the tolerance against hypoxia by increasing proline, soluble protein content, CAT, SOD, SAMDC activity, IAA and ABA concentrations, up-regulating expression of auxin-related genes (*ARF1*, *AUX1*, *AUX2*, *AUX3* and *AUX4*), reducing ethylene emission, the activity of ethylene-related enzymes and genes (*ACS* and *ACO*), and decreasing H_2_O_2_ content, O_2_^·−^ production rate and NR activity, thereby enhancing the ability of *P. praecox* to maintain the stability of cell membrane structure. These results supported our hypothesis that exogenous Spd can alleviate growth inhibition and oxidative damage of *P. praecox* under soil hypoxia stress and the cross-talk between Spd and hormones triggers the expression of related genes, and initiates downstream protective mechanisms. Overall, Spd could increase *P. praecox* adaptability to flooding stress that may be useful for the sustainable production of bamboo in practice. 2 mM Spd is more effective in relieving flooding, which can be sprayed at this concentration in the field under flooding condition.

## Materials and methods

### Plant material and experimental design

The annual seedlings of *P. praecox* were selected as experiment materials. In May 2020, the rhizome of *P. praecox* was completely taken out from the Panmugang base of Zhejiang Agriculture and Forestry University, China. The formal identifications of all samples were undertaken by Professor Renyi Gui (Zhejiang Agriculture and Forestry University). The voucher specimens were deposited at the herbarium of Zhejiang Agriculture and Forestry University (Hangzhou, China) under deposition number of 08,003. The selected bamboo seedlings were same in size and height, and had a complete structure. *P. praecox* has three to four young shoots and healthy roots. The seedlings were washed with tap water, and then rinsed twice with distilled water. Each seedling was transplanted into a plastic flowerpot with a height of 22 cm, diameter of 23 cm, and a hole at the bottom. A tray was placed at the bottom of the flowerpot. The soil used in the plastic pot was a mixture of 75% garden soil and 25% nutrient substrate. Basic soil information is as follows: pH 4.55, total nitrogen 1.58 g/kg, NH_4_-N, 7.43 mg/kg, NO_3_-N 33.60 mg/kg, total phosphorus 0.65 g/kg, available phosphorus 128.33 mg/kg; total potassium 16.38 g/kg, available potassium 705.28 mg/kg. The flowerpots were transferred to the greenhouse (N 30°23′, E 119°72′) of Zhejiang Agriculture and Forestry University with controlled conditions. The temperature in the greenhouse was controlled at 25–30 °C/15–18 °C for day/night, and the humidity was controlled at 60–75%. Half-strength nutrient solution was applied at the recovering stage. After one month, all bamboo leaves expanded and were prepared for further tests. The plant materials were selected with permission from the Panmugang Forest Farm; no ethics approvals were required.

Uniformly-grown bamboo seedlings were subjected to stress treatment, with the experiments adopting a completely random design combination. There were four treatments (1) CK (control); (2) flooding; (3) flooding + 1 mM Spd; (4) flooding + 2 mM Spd. 1 mM Spd: Put 157 ul Spd in 1L water. 2 mM Spd: put 314 ul Spd in 1L water. Each concentration is configured with 6 L per day, in order to ensure that the amount of solution sprayed per seedling is 200 mL per day. Each treatment had five replicates, and each replicate had six seedlings. The flooding treatment ensured that the water depth was 5 cm on the soil surface. From the first day after flooding, Spd was sprayed every day. The spraying time was between 09:00–10:00 to guarantee the leaves were wet without dripping. The control treatment was sprayed with distilled water under the same conditions. Seedling samples in five replicates (each replicate had six seedlings) were collected on day 0, and on the second, fourth, sixth and eighth day after the experiments were performed. The collected samples were immediately frozen in liquid nitrogen and stored at –80 °C for further use. Leaf length and area were measured after eight days of experimentation.

### Sample analysis

#### Proline content

0.5 g of fresh leaves was homogenized in 3% sulphosalicyclic acid, centrifuged at 11,500 g. Supernatant was mixed with acid ninhydrin, glacial acetic and phosphoric acid; incubated at 100 °C and cooled, toluene was added; chromophore containing toluene was read at 520 nm [85].

#### Malondialdehyde (MDA) content measurement

Lipid peroxidation is frequently expressed as MDA content. In brief, 0.5 g of fresh leaves were homogenized in 10 ml of 10% trichloroacetic acid (TCA), and centrifuged at 5000 g for 10 min. 2 ml of 0.6% thiobarbituric acid (TBA) in 10% TCA was added to an aliquot of 2 ml of supernatant. The mixture was heated in boiling water for 15 min, and then quickly cooled in an ice bath. The absorbance of the solution was measured at 440 nm, 532 nm, and 600 nm in a spectrophotometer, 3 biological replicates. MDA content was calculated with the OD absorbance. The MDA content was determined as described by Hodges et al. [86].

#### Enzyme activity assay

Fresh leaves (1 g) were ground with liquid nitrogen and suspended in 9 ml physiological saline in a pre-chilled mortar and pestle, placed in an ice bath. The homogenate was centrifuged at 12 000 × *g* for 10 min at 4 °C, and the supernatant was collected. Hydrogen peroxide (H_2_O_2_), superoxide radical (O_2_^·−^) production rate, soluble protein, catalase (CAT), superoxide dismutase (SOD), and nitrate reductase (NR) activities were determined using the assay kits supplied by the manufacturer [H_2_O_2_ assay kit (A064-1–1), O_2_^·−^ assay kit (A052-1–1), soluble protein assay kit (A045-4), CAT assay kit (A007-1–1), SOD assay kit (A001-3) and NR assay kit (A096-1–2); Nanjing Jiancheng Bioengineering Institute, China)].

ACC, ACS and SAMDC activities were determined using the ELISA plant assay kit following the manufacturer’s instructions (Shanghai Enzyme-linked Biotechnology Co., Ltd., China). The kit uses double-ant ibody one-step sandwich enzyme-linked immunosorbent assay (ELISA). Purified plant ACC antibody, ACS antibody, SAMDC antibody with HRP enzyme-catalyzed label and the corresponding enzymes were used to form an antibody-antigen-enzyme-antibody complex which produced a blue substance after reacting with the tetramethylbenzidine (TMB) substrate solution. The OD value was measured at a wavelength of 450 nm with a microplate reader to calculate the sample activity.

#### Hormone measurement

The endogenous hormones including auxin (IAA) and abscisic acid (ABA) concentrations were determined using the manufacturer’s instructions (ELISA plant hormones assay kit, Shanghai Enzyme-linked Biotechnology Co., Ltd.). Purified plant IAA antibody, ABA antibody with horseradish peroxidase (HRP) enzyme-catalyzed label and hormones were used to form an antibody-antigen-enzyme-antibody complex, which produced a blue substance after reacting with the TMB substrate solution. The OD value was measured at a wavelength of 450 nm with a microplate reader for calculation of the concentrations.

After eight days, a total of 120 leaves samples (4 treatments × 5 replicates × 6 seedlings) were placed in 15-mL glass vials containing 1 mL 0.6% water agar then immediately sealed with a gas-proof septum according to Wu [87]. After 4-h incubation in the dark at 30 °C, 1 mL of the gas was drawn from the airspace of each vial using a gas-tight syringe (Focus GC, Thermo, Massachusetts, USA) and injected into a gas chromatograph (Focus GC, Thermo) equipped with a capillary column (CP-CarboPLOT P7, California, USA) and flame-ionization detector for ET determination. The production of ethylene was then calculated based on the fresh weight (FW/t) of the samples [88].

#### RNA extraction and quantitative RT–PCR (qRT–PCR) analyses

Gene expression was measured by using quantitative reverse transcriptase polymerase chain reaction (qRT–PCR). Total RNAs in leaves were extracted using OminiPlant RNA Kit (CWBIO, CW2598, China). The concentration of mRNA was determined by a nucleic acid analyzer (Nano Drop 2000c, Thermo Scientific, USA) and the RNA quality was assessed using agarose gel electrophoresis. The extracted RNA was reverse transcribed into cDNA using the PrimeScript™ RT reagent Kit with gDNA Eraser (Takara, RR047A, China) and the synthesized cDNA was subjected to PCR. Primers were designed for the sequences of *Actin*, *ARF1*, *ACS*, *ACO*, *AUX1*, *AUX2*, *AUX3* and *AUX4* by Primer 5.0. *Actin* was used as a control, and used for qRT–PCR (Table 3). The primer length was set to between 18 and 26 bp, and the expected length of the amplified product was between 80 and 250 bp. The primers were synthesized by Sangon Biotech (Shanghai, China). The fluorescent dye used for qRT–PCR (Roche Light Cycler 480II, Switzerland) was Ultra SYBR Mixture (Takara, RR820A, China). Relative gene expression was measured by the 2^−∆∆CT^ method, relative to the house-keeping gene *Actin* [89].

**Table 3:** qRT-PCR specific primers for examined genes

Gene name	Primer sequence (5’-3’)	Accession (http://www.ncbi.nl.nih.gov/)
*Actin*	F: CGTCAAAGCCCCAAGAACAC R: GCTAGGAAAGACAGCCCTGG	FJ601918.1
*ARF1*	F: ATGCACCTGGTATGGCGAAT R: TCCAAGTGACCATGCCCAAA	KU721918.1
*ACS*	F: TCAGCTCGTTCGTCCATCAC R: TTAGCTACGCGTTGGTCGTC	AB085172.1
*ACO*	F: GATCACCAACGGCAGGTACA R: TCCTCGAACACGAACTTGGG	AB044747.1
*AUX1*	F: GTTCGTGAAGGTGAGCATGG R: CGTTCATGCCGTTCATCCCT	KU721904.1
*AUX2*	F: TCTGAGGATGTACGGAGGGT R: GCATCAGATCGCCGTCCTTG	KU721905.1
*AUX3*	F: AAGGGCATGAACGAGAGCAA R: CGACTCGACGAACATCTCCC	KU721906.1
*AUX4*	F: TGACCAGCCGATGACGAAG R: GCTGCTTGGAAGGTGTTCCT	KU721907.1

### Statistical analysis

The physiological parameters were analyzed statistically via two-way analysis of repeat measurements (ANOVA) according to Duncan’s Multiple Range test. The F value was obtained from ANOVA. In the text, ns, *, ** and *** represent not significant, and significant difference at *P* < 0.05, *P* < 0.01, and *P* < 0.001, respectively. The sample variability was presented in a line diagram with SD. The significant differences among treatments were marked by different superscripts (a-d) at the level of *P* < 0.05 by Duncan’s method. All data were analyzed using the SPSS software package (SPSS 20.0, IBM company, USA).

## Data Availability

All relevant data are within this article and its Additional files. The datasets used and/or analysed during the current study are available from the corresponding author on reasonable request.

## Abbreviations

Spd: Spermidine
CAT: Catalase
SOD: Superoxide dismutase
SAMDC: S-adenosylmethionine decarboxylase
ABA: Abscisic acid
IAA: Indole-3-acetic acid
H_2_O_2_: Hydrogen peroxide
O_2_: Superoxide radical
ACO: ACC oxidase
ACS: ACC synthase
ARF1: Auxin responsive factor1
AUX1: Auxin1 protein
AUX2: Auxin2 protein
AUX3: Auxin3 protein
AUX4: Auxin4 protein
Pas: Polyamines
Put: Putrescine
Spm: Spermine
SAM: S-adenosyl-L-methionine
ADC: Arginine decarboxylase
NO: Nitric oxide
LL: Leaf length
LA: Leaf area
NR: Nitrate reductase;
O_2_: Oxygen
NOS: NO synthase
MeJA: Methyl jasmonate
SA: Salicylic acid
GA3: Gibberellins
ZR: Zeatin-riboside
Aux/IAA: Auxin/indole-3-Acetic Acid
ARFs: AUXIN RESPONSE FACTORS
AuxREs: Auxin response elements
TCA: Trichloroacetic acid
TBA: Thiobarbituric acid
ELISA: Enzyme-linked immunosorbent assay
TMB: Tetramethylbenzidine
HRP: Horseradish peroxidase
